# Levels of Adipokines in Amniotic Fluid and Cord Blood Collected from Dichorionic-Diamniotic Twins Discordant for Fetal Growth

**DOI:** 10.1371/journal.pone.0154537

**Published:** 2016-05-02

**Authors:** Seung Mi Lee, Joong Shin Park, Errol R. Norwitz, Sitthysack Panyavatthanasinh, Sun Min Kim, JoonHo Lee, Chan-Wook Park, Byoung Jae Kim, Jong Kwan Jun

**Affiliations:** 1 Department of Obstetrics & Gynecology, Seoul National University College of Medicine, Seoul, Korea; 2 Department of Obstetrics & Gynecology, Tufts University School of Medicine, Boston, Massachusetts, United States of America; 3 Department of Obstetrics & Gynecology, University of Health Sciences, Vientiane, Lao PDR; 4 Department of Obstetrics & Gynecology, Seoul Metropolitan Government Seoul National University Boramae Medical Center, Seoul, Korea; SERGAS (Servizo Galego de Saude) and IDIS (Instituto de Investigación Sanitaria de Santiago), the NEIRID Lab, Research Laboratory 9, Santiago University Clinical Hospital. Santiago de Compostela, SPAIN, SPAIN

## Abstract

**Objective:**

To compare the concentrations of adipokines in amniotic fluid (AF) and cord blood collected from discordant dichorionic-diamniotic (DCDA) twin fetuses.

**Study Design:**

The study population included DCDA twins discordant for fetal growth (birth weight difference >10%) who either underwent mid-trimester amniocentesis for routine clinical indication (Cohort 1) or whose amniotic fluid was collected at the time of delivery (Cohort 2). In both cohorts, cord blood was collected at delivery.

**Results:**

A total of 92 twin pairs were enrolled (n = 49 in Cohort 1; n = 43 in Cohort 2). In Cohort 1, the concentrations of adiponectin (median, 68.5 ng/mL vs 61.4 ng/mL; p<0.05) and leptin (median, 13.9 ng/mL vs 11.2 ng/mL; p<0.1) in mid-trimester AF were significantly higher in smaller compared with larger twins. In Cohort 2, the concentration of serpin E1 (median, 246.0 ng/mL vs 182.8 ng/mL; p<0.01) in AF at delivery was significantly higher in smaller twins, but no difference was noted in adiponectin and leptin concentrations. Levels of adiponectin (median, 10425.5 ng/mL vs 11552.0 ng/mL; p<0.005) and leptin (median, 2.1 ng/mL vs 2.6 ng/mL; p<0.005) were significantly lower in the cord blood of smaller twins whereas cord blood concentrations of serpin E1 (median, 15.5 ng/mL vs 13.3 ng/mL; p<0.05) was higher in the smaller twins.

**Conclusion:**

In discordant DCDA twin pairs, concentrations of adiponectin, leptin, and serpin E1 in mid-trimester AF, AF at delivery, and cord blood at birth vary significantly but predictably between the smaller and larger twins.

## Introduction

The intrauterine environment is known to be a critical determinant of adult health. For example, fetal growth restriction (FGR) is associated with the development of adult metabolic diseases, such as obesity, diabetes, hypertension, and coronary heart disease [1]. In the setting of a suboptimal intrauterine environment, the growth restricted fetus undergoes a series of developmental adaptations including a permanent change in its hypothalamic-pituitary-adrenal axis and in insulin-glucose metabolism. It is these changes that appear to be responsible for the increased risk of metabolic diseases in adulthood [2–4].

Adipose tissue is an endocrine organ capable of modulating metabolic and inflammatory processes and contributing to the development of metabolic complications [5]. It has been suggested that adipose tissue may provide the critical link between FGR and subsequent adult diseases [6–9]. Fetuses with growth restriction have a reduced overall body fat mass, but a disproportionate increase in visceral fat tissue [7]. This central obesity has been associated with rapid catch-up growth and the early development of insulin resistance [6–8].

Adipose tissue affects metabolism and energy homeostasis throughout the body primarily through the secretion of adipokines, such as leptin and adiponectin. Given the critical role that adipose tissue plays in fetal growth and the risk of subsequent metabolic diseases, it is important to understand the association between adipokine concentrations within the various intrauterine compartments and fetal growth, especially in the setting of FGR. Unfortunately, the literature on this topic is conflicting. Several investigators have reported a decrease in cord blood concentrations of leptin and adiponectin in growth-restricted fetuses [10–14], whereas others found no association [15–17]. In addition, there are few studies on adipokine concentrations in the amniotic fluid (AF) or on longitudinal changes throughout pregnancy.

In this study, we compare the concentrations of a series of adipokines in AF and cord blood at birth between the larger and smaller fetuses in a cohort of dichorionic-diamniotic (DCDA) twin pairs discordant for fetal growth. By focusing on twins, we hope to abrogate the effects of maternal and intrauterine variations on fetal growth. In addition, we measured adipokine concentrations in mid-trimester AF to examine the longitudinal change in AF adipokine concentrations.

## Materials and Methods

### Study design

This retrospective cohort study consisted of consecutive twin pregnant women cared for at Seoul National University Hospital with the following inclusion criteria: (1) DCDA twins with birth weight discordancy (birth weight difference greater than 10% between the larger and smaller twin); (2) AF retrieved at mid-trimester or at birth; (3) cord blood collected at birth; and (4) who delivered in our institution. Cases were classified into Cohort 1 or Cohort 2 depending on the timing of AF retrieval. Cohort 1 included women who underwent clinically-indicated mid-trimester amniocentesis between 15–20 weeks of gestation for fetal karyotyping; Cohort 2 consisted of women whose AF and cord blood was collected at the time of cesarean delivery. Cases with early preterm delivery (<32 weeks of gestation), major fetal anomalies, or intrauterine fetal death were excluded.

Clinically-indicated mid-trimester amniocentesis was performed on each twin under ultrasound guidance after informed consent had been obtained. Amniocentesis at the time of cesarean delivery was performed just before amniotomy by inserting a needle into the fetal membranes of each twin under direct visualization after performing the hysterotomy. Cord blood was collected after delivery of the neonates by withdrawing blood from the umbilical vein of each twin. Retrieved AF and cord blood was centrifuged, aliquoted, and stored at -70°C until assayed.

The Institutional Review Board at Seoul National University Hospital approved the study. Patients provided written consent for the collection and use of these samples and their accompanying clinical information for research purposes.

### Measurement of adipokines

Stored AF (mid-trimester AF in Cohort 1; AF at birth in Cohort 2) and cord blood samples were analyzed for multiple adipokines and obesity-related biomarkers (Complement Factor D/Adipsin, Serpin E1/PAI-1, Adiponectin/Acrp30, C-Reactive Protein, CCL2/MCP-1, Leptin, Resistin) using the Luminex Performance Assay kit (R&D Systems, Inc., Minneapolis, MN, USA). For multiplex assays, the analyte-specific antibodies were pre-coated onto color-coded microspheres and the immobilized antibodies were allowed to bind the analyte of interest. After adding a biotinylated antibody cocktail specific to the analytes of interest, streptavidin-phycoerythrin conjugate bound to the captured biotinylate antibodies were read with the Luminex analyzer. The magnitude of the phycoerythrin-derived signal is directly proportional to the amount of analyte bound. For analysis, values below the lower limit of detection for each analyte were recorded as the lower limit of quantification (LLOQ).

### Statistical methods

The clinical characteristics between the large and small twin pairs were compared with Wilcoxon signed rank test for continuous variables and with McNemar test for categorical variables. The concentrations of analytes in AF and cord blood between large and small twins were also compared with Wilcoxon signed rank test. A generalized estimating equation (GEE) was adopted for the adjustment of confounding variables in the relationship between twin birth weight order and analyte concentrations. GEE can be used in multivariate analysis from the same subject [18,19] and in family-based association studies [20]. Statistical analyses were conducted using the IBM SPSS version 20. *P*<0.05 was considered significant.

## Results

### Clinical characteristics

A total of 92 twin pairs met the inclusion criteria, including 49 cases in Cohort 1 and 43 cases in Cohort 2. Table 1 shows the clinical characteristics of the twin pairs. The fetal biometric measurements at the time of mid-trimester amniocentesis were not significantly different between the twin destined to be smaller at birth and the twin destined to be larger at birth, although birth weights were significantly different between twin pairs in both cohorts.

**Table 1:** Clinical characteristics of the study population.

**Cohort 1 (Mid-trimester amniocentesis; n = 49)**			
	Small twin	Large twin	p
Maternal age at amniocentesis (years)	34 (33–36)		
Nulliparity (n)	40 (82%)		
Gestational age at amniocentesis (weeks)	17 (16.4–17.8)		
Indication for amniocentesis (n)			
Advanced maternal age	36 (74%)		
Abnormal serum screening	10 (20%)		
Maternal request	3 (6%)		
Biparietal diameter at amniocentesis (cm)*	3.7 (3.6–3.95) (n = 41)	3.8 (3.5–4.05) (n = 41)	NS
Femur length at amniocentesis (cm)*	2.15 (2.0–2.4) (n = 42)	2.2 (2.0–2.4) (n = 42)	NS
Gestational age at delivery (weeks) *	37.1 (36.0–37.9)	37.1 (36.0–37.9)	NS
Presenting twin (n)^†^	26 (53%)	23 (47%)	NS
Neonatal sex (male) (n)^†^	20 (41%)	27 (55%)	NS
Birth weight (grams) *	2240 (1875–2490)	2660 (2310–2935)	<0.001
Small for gestational age at delivery (n)^†^	31 (63%)	5 (10%)	<0.001
Cesarean delivery (n)^†^	35 (71%)	35 (71%)	NS
**Cohort 2 (Amniocentesis at delivery; n = 43)**			
	Small twin	Large twin	p
Maternal age at amniocentesis (years)	32 (30–35)		
Nulliparity (n)	35 (81%)		
Gestational age at amniocentesis (weeks)	37.0 (35.3–37.7)	37.0 (35.3–37.7)	NS
Gestational age at delivery (weeks) *	37.0 (35.3–37.7)	37.0 (35.3–37.7)	NS
Presenting twin (n)^†^	12 (28%)	31 (72%)	NS
Neonatal sex (male) (n)^†^	19 (44%)	25 (58%)	NS
Birth weight (grams) *	2150 (1850–2500)	2690 (2340–3040)	<0.001
Small for gestational age at delivery (n)^†^	30 (70%)	0 (0%)	(-)
Cesarean delivery (n)^†^	43 (100%)	43 (100%)	NS

Data are presented as median and interquartile range; NS, not significant

* Analyzed with Wilcoxon signed rank test

^†^ Analyzed with McNemar test

### Concentrations of biomarkers in AF and cord blood

Table 2 compares the concentrations of adipokines and obesity-related biomarkers in AF and cord blood between the small and large twin pairs. In mid-trimester AF, the concentrations of adiponectin, leptin, and C-reactive protein of twins destined to be smaller at birth were significantly higher than twins destined to be larger. The difference in concentrations of adiponectin and leptin between twin pairs remained significantly different after adjustment for fetal sex and birth order. However, the concentrations of adiponectin, leptin, and C-reactive protein in AF collected at the time of birth were not significantly different between twin pairs. The median concentration of serpin E1 in the AF of smaller twins were significantly higher than that in the AF collected from the larger twin, and this difference remained significant after adjustment (Table 2).

**Table 2:** Concentrations of select chemokines in smaller versus larger twins.

**Mid-trimester AF, Cohort 1 (Mid-trimester amniocentesis; n = 49)**			
	Small twin	Large twin	P value*
Adiponectin (ng/mL)	**68.5 (40.1–106.6)**	61.4 (41.7–77.7)	<0.05†
Leptin (ng/mL)	**13.9 (9.9–25.3)**	11.2 (7.5–18.9)	<0.01†
C-Reactive Protein (ng/mL)	**25.2 (12.5–57.4)**	18.1 (6.4–54.9)	<0.005
Complement factor D (ng/mL)	1256.2 (927.9–1793.3)	1190.7 (872.8–1653.5)	NS
Serpin E1 (ng/mL)	174.3 (105.9–230.2)	161.3 (110.6–250.1)	NS
CCL2/MCP-1 (ng/mL)	0.57 (0.45–0.77)	0.54 (0.38–0.71)	NS
Resistin (ng/mL)	12.7 (7.3–23.6)	14.3 (6.9–25.1)	NS
**AF at the time of delivery, Cohort 2 (Amniocentesis at delivery; n = 43)**			
	Small twin	Large twin	P value*
Adiponectin (ng/mL)	49.7 (36.9–74.7)	56.7 (39.4–75.5)	NS
Leptin (ng/mL)	**2.0 (1.3–3.1)**	2.0 (1.0–2.6)	NS
C-Reactive Protein (ng/mL)	26.3 (9.8–62.7)	25.1 (11.1–73.7)	NS
Complement factor D (ng/mL)	**878.3 (656.2–1163.5)**	868.0 (650.6–1150.9)	NS
Serpin E1 (ng/mL)	**246.0 (167.7–290.9)**	182.8 (150.1–249.9)	<0.01†
CCL2/MCP-1 (ng/mL)	0.32 (0.21–0.44)	0.33 (0.26–0.50)	NS
Resistin (ng/mL)	12.3 (9.1–20.2)	12.3 (8.7–21.3)	NS
**Cord blood at the time of delivery, Cohort 1 and 2 (n = 92)**			
	Small twin	Large twin	P value*
Adiponectin (ng/mL)	10425.5 (7378.4–13183.0)	**11552.0 (9351.7–14287.8)**	<0.005†
Leptin (ng/mL)	2.1 (1.1–4.2)	**2.6 (1.4–4.8)**	<0.005†
C-Reactive Protein (ng/mL)	**26.6 (16.4–52.5)**	20.1 (14.9–44.6)	<0.001
Complement factor D (ng/mL)	5183.8 (4427.5–5888.1)	**5364.0 (4653.2–6260.5)**	0.083
Serpin E1 (ng/mL)	**15.5 (9.7–34.0)**	13.3 (9.7–22.5)	<0.05†
CCL2/MCP-1 (ng/mL)	0.13 (0.08–0.17)	0.12 (0.10–0.17)	NS
Resistin (ng/mL)	69.3 (38.6–193.0)	84.0 (35.5–163.6)	NS

Data are presented as median and interquartile range; NS, not significant

* Analyzed with Wilcoxon signed rank test

^†^ Significant after adjustment for fetal sex and birth order

In all 92 twin pairs from both cohorts, the concentrations of adiponectin and leptin in the cord blood collected from the smaller twins were significantly lower than that collected from the larger twin, whereas the cord blood concentrations of C-reactive protein and serpin E1 in the smaller twin were significantly higher than that of larger twin (Table 2). This difference in cord blood concentrations of adiponectin, leptin and serpin E1 remained significant after adjustment.

## Discussion

### Main findings

The principal findings of this study were: (1) concentrations of adiponectin and leptin in mid-trimester AF of twins destined to be smaller at birth were significantly higher than twins destined to be larger; (2) concentrations of adiponectin and leptin in AF at the time of delivery were not significantly different between smaller and larger twin pairs, but the concentration of serpin E1 in AF at the time of delivery was significantly higher in smaller twins than in larger twins; and (3) levels of adiponectin and leptin in cord blood of smaller twins were significantly lower than that in the cord blood of the larger twins, whereas the concentration of serpin E1 in cord blood of smaller twins was higher than that in larger twins.

### Leptin and adiponectin in cord blood of growth restricted fetuses

Leptin is a circulating hormone that affects energy homeostasis by regulating energy expenditure and food intake [21]. Levels of leptin are elevated in the circulation of adults with obesity, metabolic syndrome, and cardiovascular disease [22, 23]. In contrast, adiponectin appears to have anti-atherogenic and anti-inflammatory properties, likely mediated by its affects on glucose and lipid metabolism. In adults, circulating levels of adiponectin are is inversely correlated with body weight or body fat mass [24, 25].

Prior studies investigating the association between leptin/adiponectin and FGR have shown conflicting results. Most reported that neonates with FGR had lower leptin concentrations than their appropriately grown controls [10–12,26], but others found no difference [15,16]. Similarly, some studies have shown a positive correlation between cord blood adiponectin concentrations and birth weight, with growth-restricted fetuses having lower adiponectin levels [13,14], but other study found no such association [17]. These inconsistent results may be due to the effect of a number of variables which could affect the cord blood concentrations of adipokines, including the gestational age at delivery, maternal obesity, or maternal diet [27]. In an effort to abrogate these maternal and intrauterine variables, we compared the concentrations of adipokines in cord blood between the larger and smaller fetuses in DCDA twin pairs discordant for growth. Our findings confirm prior reports [10–14,26] that both leptin and adiponectin concentrations are decreased in the cord blood of smaller fetuses. Cord blood leptin is derived from a number of sources, including maternal adipose tissues, fetal adipose tissue, and the placenta [28]. Lower concentrations of adipokines in growth-restricted fetuses may be attributed to reduced fat mass or reduced placental production. The studies by Kamoda et al. [29] and Takaya et al. [30] showed that growth-restricted neonates had lower concentrations of adiponectin and that this was associated with the future development of insulin resistance and metabolic complications.

### Serpin E1 in fetal growth restriction

Serpin E1, also known as plasminogen activator inhibitor-1 (PAI-1), regulates fibrinolysis and is associated with endothelial dysfunction. Serpin E2 is derived from endothelial cell and adipose tissue, and is believed to regulate insulin resistance. The literature suggests that, in adults, high levels of serpin E1 is associated with dyslipidemia and metabolic syndrome [31]. Serpin E1 levels are known to be elevated in the maternal circulation of pregnancies with FGR and uteroplacental insufficiency [32], but there are few studies on the relationship between serpin E1 and growth restriction in the fetus or neonate. In the study of Boutsikou et al. [33], circulating levels of serpin E1 were not significantly different between growth-restricted neonates and neonates with normal growth. In the current study, smaller fetuses had higher concentrations of serpin E1 in both cord blood and AF at delivery, which is consistent with the result of studies in adults with metabolic syndrome [31].

### Adipokines in amniotic fluid of growth-restricted fetuses

In the current study, the pattern of adipokine concentrations were different between AF collected in the mid-trimester and AF collected at delivery. In the mid-trimester, AF leptin and adiponectin concentrations were higher in the twin destined to be smaller at birth than in the twin destined to be larger, and the concentration of serpin E1 was not significantly different between the twins. However, at the time of birth, the AF leptin and adiponectin concentrations were not different between the twin pairs discordant for growth, whereas the concentration of serpin E1 was higher in the smaller twins than the larger twins.

To our knowledge, this is the first study to compare the concentrations of adipokines in twin AF at the time of birth and also the first study to investigate the temporal change in AF adipokine concentrations during pregnancy and its association with fetal growth. There are two possible explanations for the temporal change in AF adipokine concentration. First, the precise source of AF adipokines is still unknown, and may be different in the mid-trimester and at the time of birth. In the mid-trimester, fetal urine is a less important constitute of AF and it is possible that the placenta or fetal membranes might be the major source of AF proteins at this time [34–36]. In support of this explanation, prior studies have shown excessive expression of leptin in placentas collected from women with preeclampsia [37,38] and elevated concentrations of leptin and adiponectin in mid-trimester AF have been measured before the development of FGR or preeclampsia in singleton pregnancies [36,39]. In third trimester, the source of AF adipokines may include both the fetus and the placenta, which may explain why AF leptin and adiponectin concentrations were not different at delivery, while serpin E1 levels were increased in the smaller twins. The second possible explanation is that placental and/or fetal expression of adipokines may be different between the mid-trimester and third trimester, resulting in temporal changes in AF concentrations. This temporal change in placental and/or fetal expression of adipokines could be only demonstrated by longitudinally sampling fetal cord blood or placental tissues or AF serially in the same patient from the mid-trimester to term, which would be difficult to do in humans.

In conclusion, concentrations of adiponectin, leptin, and serpin E1 vary significantly between smaller and larger twins, and show different patterns of change in mid-trimester AF, AF at delivery, and cord blood at birth in DCDA twin pairs discordant for growth. Additional studies are needed to determine whether these changes are causally related to the development of FGR and/or to the subsequent development of metabolic complications known to occur at a higher incidence in growth-restricted fetuses.
